# Cold chain management practices of non-vaccine commodities in public health facilities of Rwenzori Region, Western Uganda

**DOI:** 10.1186/s40545-023-00516-5

**Published:** 2023-01-18

**Authors:** Yona Tumwine, Kalidi Rajab, Paul Kutyabami, Jean Baptiste Nyandwi, Domina Asingizwe

**Affiliations:** 1 EAC Regional Centre of Excellence for Vaccines, Immunization, and Health Supply Chain Management, College of Medicine and Health Sciences, University of Rwanda, Kigali, Rwanda; 2 National Medical Stores, Kampala, Uganda; 3 College of Health Sciences, Makerere University, Kampala, Uganda

**Keywords:** Cold chain, Non-vaccine commodities, Cold chain management, Uganda

## Abstract

**Background:**

Despite the evidence that cold chain management practices affect the potency and effectiveness of both vaccines and non-vaccine commodities, most of the researches in Uganda focus on vaccines. This study assessed the cold chain management practices of non-vaccine cold chain commodities in public health facilities of the Rwenzori Region, Uganda.

**Methods:**

A cross-sectional study was conducted in a random sample of 69 level III and IV health facilities. The respondents were store managers at health facilities. Data on stock and storage management practices and level of knowledge on cold chain management were assessed. Data were collected and entered into Microsoft excel 2017, cleaned, and later exported into IBM SPSS version 26 for analysis. The management practices were graded as poor (< 50% score), fair (50–75% score), or good (> 75% score).

**Results:**

Results from the 69 facilities indicated that the stock management practices were graded as fair for lesser than half of the facilities 28 (40.6%). Few facilities were correctly filling stock cards 20 (29%) and conducting physical inventories 19 (27.5%). The refrigerator storage management practices were fair for nearly half of the facilities 32 (46.4%). Among the facilities that had a refrigerator 53 (76.8%), 39 (70.9%) utilized it for storing both vaccines and non-vaccine commodities. The cold chain management practices at service delivery points were fair for 32 (46.4%) health centers. A larger proportion of the participants 69 (65%) had knowledge of cold chain storage. Most of the participants 47 (67.8%) were knowledgeable about the heat sensitivity of the cold chain commodities, however, almost half (48.1%) of them lacked knowledge on refrigerator use.

**Conclusion:**

The management practices for non-vaccine cold chain commodities in health centers were fair. More than a third of the facility store managers lacked knowledge on cold chain management practices for non-vaccine commodities. There is a need to strengthen the capacity of the facilities’ store managers and provide equipment specific for non-vaccine cold chain commodities.

## Background

The success of health service delivery depends on the availability of medicines and vaccines at health facilities and the protection of their potency [1]. The quality of cold chain commodities can only be assured by a functional cold chain system [2]. All over the world, the importance of the cold chain is apparent to governments and key stakeholders within the health sector. However, there are less significant efforts put to appropriately manage the supply chain logistics such as transport, storage, packaging, technical capacity and many other sensitive activities that help to maintain the safety and quality of such cold chain [3]. The Center for Disease Control (CDC) estimates that each year, £300 m worth of cold chain medicines alone are destroyed globally due to improper storage and distribution [4]. The World Health Organization (WHO) estimates also indicate that poor medicine management systems account for 70% of inefficiencies in the health sector with a significant portion of these inefficiencies associated with storage related challenges, especially in Africa and Asia [4]. Such a break in the cold chain not only wastes money but, more importantly, jeopardizes the quality of the diagnosis and treatment [5].

A number of factors affect cold chain systems. These include breakdown of refrigerators and freezers, delays during transportation, inappropriate refrigerators, long duration of storage at the health unit, improper use of refrigerators, power interruptions, and lack of trained personnel capable of managing the cold chain [6]. In addition, non-uniformity in storage temperature instructions on the label, patient education and lack of awareness about cold chain management are responsible for the breakdown in the cold chain management [4]. Studies in Uganda found that cold chain systems are struggling to effectively support national immunization programs in ensuring the availability of safe and potent vaccines and cold chain storage [7]. Transport and storage equipment for most health facilities was either lacking or obsolete resulting in frequent stock outs and a reduction in the potency of the supplied vaccines. Domestic refrigerators are commonly used yet not safe for vaccine storage [8, 9]. Little is known about the management practices of non-vaccine cold chain commodities and is likely to be poor given the lack of focus on these commodities [7]. Poor cold chain management practices of non-vaccine cold chain commodities may lead to loss of potency and financial resources, ultimately affecting the quality of diagnosis and treatment [10].

Despite the evidence that cold chain management practices affect the potency and efficacy of both vaccines and non-vaccine commodities, most of the support towards effective management and researches in Uganda are focused only on vaccines [8]. In addition, cold chain technicians at the district level have concentrated on the cold chain of vaccines with minimal emphasis on non-vaccine commodities’ cold chain. Strikingly, Non Expanded Program on Immunization (EPI) cold chain commodities such as laboratory reagents, insulin injection, oxytocin among others are stuffed with vaccines [9]. Since non-vaccine cold chain commodities form a core part of diagnostic and curative interventions, their protection against loss of potency and efficacy is of great importance. Thus, research leading to improvement in their management is paramount. This study therefore assessed the cold chain management practices of non-vaccine commodities in Public health facilities of Rwenzori Region, Western Uganda. The findings of this study provide guidance to different stakeholders at policy and managerial levels on possible interventions to improve cold chain management of non-vaccine health commodities.

## Methods

### Study design, setting and population

This was a descriptive cross-sectional study employing quantitative approach of data collection. Cold chain management practices, the level of knowledge of staff on cold chain management, and factors affecting cold chain management practices of non-vaccine commodities were determined. The study was conducted in public Health Centers (HCs) located in the Rwenzori sub-region. Rwenzori sub-region is one of the three (3) sub-regions in Western Uganda and comprises nine (9) districts with 135 level II, 84 level III and 13 level IV Health Centers (HC), 4 General Hospitals (GHs) and 1 Regional Referral Hospital (RRH). The study was conducted in level III and IV HCs (69 in total) from all districts because they are widely spread, serve a bigger population number and have limited pharmaceutical technical human resource, hence were more likely to experience issues on cold chain management. The study population included healthcare workers involved in the management of non-vaccine cold chain commodities in the selected level III and IV health centers.

### Sample size and sampling method

*Health facilities:* The sample size for the facilities was obtained using Yamane’s formula [11]:

where *n* is the sample size, *N* is the population size, which is the number of health facilities (= 97) in this study and e is the margin of error (5%). *n* = 78 health facilities.

The sample size composed of 68 level III HCs and 10 level-IV HCs proportionately determined as shown in Table 1. The HCs were selected by simple random sampling within each district. Microsoft Excel 2017 was used to generate the random list of facilities.

**Table 1:** Sample size for the health facilities

Districts	Number of HCIII	Sample of HCIII	Number of HCIVs	Sample of HCIV	Total facilities sampled
Kabarole	15	12	3	2	14
Kyenjojo	10	8	1	1	9
Kamwenge	8	6	1	1	7
Kasese	24	19	1	1	20
Bundibugyo	7	6	2	1	7
Bunyangabu	7	6	1	1	7
Kitagwenda	4	3	1	1	4
Ntoroko	2	2	2	1	3
Kyegegwa	7	6	1	1	7
Total	84	68	13	10	78

*HCIII* Level III Health Centers, *HC IV* Level IV Health Centers

*Respondents:* The study included one respondent directly involved in cold chain management (store persons) at health center, totalling 78 respondents. The stock persons were found to be the best to answer the questions for this study because these are the ones managing stock on daily basis.

### Data collection

In this study, two data collection instruments were used; an observation checklist and a questionnaire. The checklist was used to obtain information about cold chain management practices and also deployed to capture data on health facility factors and characteristics including information on health system factors. The questionnaire captured data on the demographic characteristics and knowledge of participants. A pilot study was conducted in two health centers (HCIII and HCIV) outside Rwenzori Region to improve question formulation and elimination of ambiguity.

### Data analysis

The data were analyzed using Statistical Package for the Social Sciences (SPSS) version 26. For the outcome variable of cold chain management practices, scores were determined. The scores were calculated numerically, where each element in the parameters assessed was given a value of one for a positive outcome or a value of zero for a negative or null outcome. The scores were then aggregated to produce a composite variable score to indicate the facilities cold chain management practices. The aggregate score of a facility was then changed to a percentage and classified as poor if it was below 50%, fair if it ranged from 50 to 75% and good if the score was above 75%. For the assessment of level of knowledge, average scores were determined using the procedure as in the case of cold chain management practices. However, here the aggregated scores produced a composite variable score to indicate the levels of knowledge among the respondents. This is widely used because of its accuracy [12]. Descriptive statistics were summarized as frequencies and percentages.

## Results

### Health facility characteristics

Nine among 78 participants did not respond to the questionnaire, therefore, 69 respondents were reached in total and the results are presented accordingly in this section.

Table 2 indicates the characteristics of health facilities. The majority 62 (90.0%) of the surveyed health facilities were level III Health centers. Most of them 36 (52.2%) were using national grid hydropower and had no backup 52 (75.4%) power supply. A significant proportion 31 (44.9%) of the health centers had no cold chain storage facility for non-vaccine supplies.

**Table 2:** Characteristics of health facilities

Variable	Categories	Frequency, *n* = 69	Percentage
Level of care	HCIII	62	90.0
	HCIV	7	10.0
Existence of SOPS	Present	19	27.5
	Absent	50	72.5
Power supply at the facility	Hydropower	36	52.2
	Generator	24	34.8
	Solar	9	13.0
Backup power supply	Present	17	24.6
	Absent	52	75.4
Existence of cold chain storage facility for non-vaccine commodities	Present	38	55.1
	Absent	31	44.9
Facility has update cold chain management policy	Present	14	20.3
	Absent	54	79.7

### Cold chain management practices for non-vaccine commodities

Cold chain management practices were assessed in three categories: stock management practices, storage management practices at health facilities, and storage management practices at service delivery points. These results are presented in the following section.

### Stock management practices

Stock management practices were graded as fair and good in 28 (40.6%) and 17 (24.6%) health centers, respectively. Only 26 (37.7%) health centers had stock cards for recording their stock movement. It is shown that 20 (29.0%) of the assessed health centers were correctly filling the stock cards Moreover, the findings revealed that only 19 (27.5%) of the health centers were conducting monthly physical inventory as shown in Table 3.

**Table 3:** Stock management practices for non-vaccine commodities cold chain

Parameter	Frequency *n* = 69	Percentage
*Stock management practices*		
Stock cards are available	26	37.7
Stock cards are correctly filled	20	29.0
Physical inventories are done monthly	19	27.5
Stock out of non-vaccine cold chain supplies	35	50.7
Adherence to FIFO-FEFO	51	73.9
*Grading of stock management practices*		
Poor < 50% score	24	34.8
Fair 50–75% score	28	40.6
Good > 75% score	17	24.6

### Non-vaccine refrigerator cold chain storage management practices at the health centers’ store

The findings showed that the practice was poor and fair in 29 (42%) and 32 (46.4%) health centers, respectively. Most of the health centers had a refrigerator 53 (76.8%), but they were using the same refrigerator 39 (70.9%) for both vaccines (EPI commodities) and non-vaccine commodities. Table 4 shows the details.

**Table 4:** Storage management practices for non-vaccine cold chain supplies at the store

Parameter	Frequency *n* = 69	Percentage
*Refrigerator storage management practices*		
Availability of non-vaccine refrigerator	53	76.8
Refrigerator in good condition	47	68.1
Same refrigerator used for both EPI and non-vaccine cold chain	39	70.9
Refrigerator well situated away from sunlight	35	50.7
Refrigerator is not overstocked	31	56.4
Correct temperature range at all times	31	44.9
Correct documentation of temperature chart	25	52.1
Refrigerator has an alarm	28	56.0
Cold chain commodities are in original packs with information leaflet	31	62.0
*Grading of refrigerator storage management*		
Poor < 50% score	29	42.0
Fair 50–75% score	32	46.4
Good > 75% score	8	11.6

### Storage practices at the service delivery points (SDP)

The cold chain management practices at service delivery points were fair in 32 (46.4%) health centers. Lesser than half of the health centers 27 (39.1%) had cooler boxes at the SDPs and records of regular checking of cooler boxes were available. More details are provided in Table 5.

**Table 5:** Storage practices of non-vaccine commodities at service delivery points

Parameters assessed	Frequency *n* = 69	Percentage
Refrigerator available at SDP	27	39.1
Adequate cold boxes at SDP	27	39.1
Cooler box temperature between 2 and 8 degrees	13	18.8
Records of regular checking of cooler boxes available	17	24.6
Commodities are correctly parked in the cooler boxes	16	23.1
Thermometer available and functional for cooler boxes	9	13
Fluid ice parks are used	18	26
Ice parks are changed on daily basis	11	15.9
*Grading of storage practices at SDP*		
Poor < 50% score	17	24.6
Fair 50–75% score	32	46.4
Good > 75% score	20	29.0

### Knowledge on non-vaccine commodities cold chain storage and handling

**Fig. 1:**
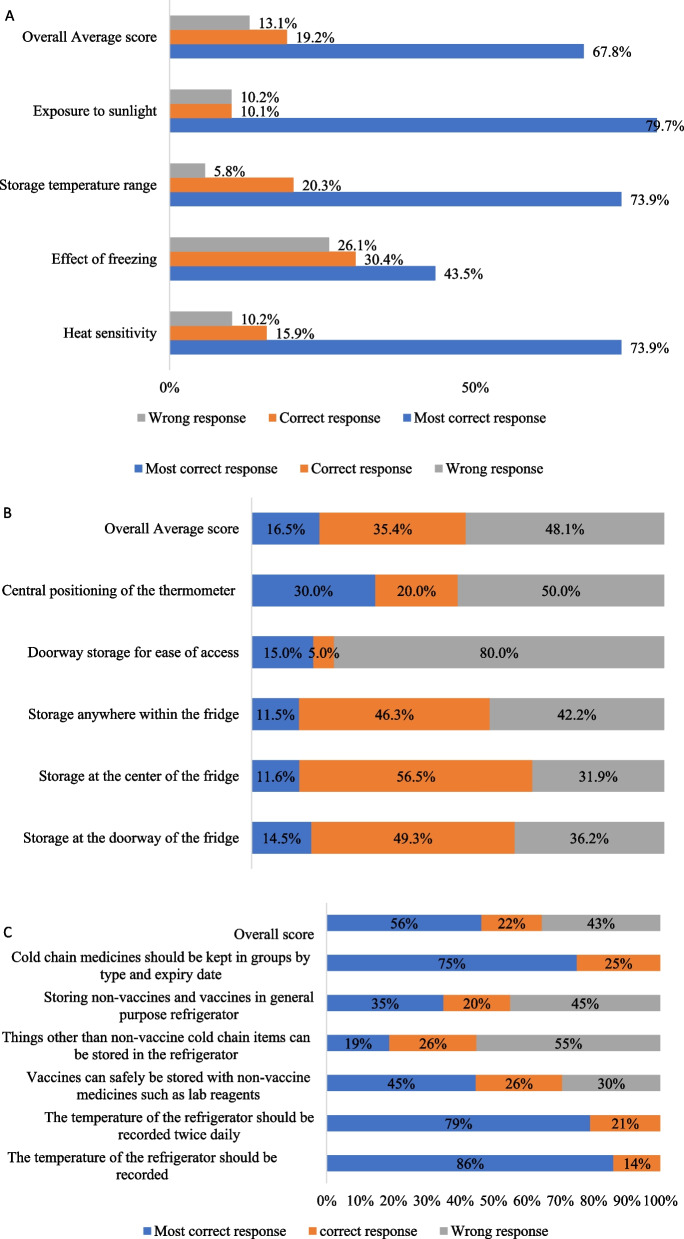
**A** Knowledge on exposure of cold chain commodities to extreme temperatures. **B** Knowledge of refrigeration equipment use. **C** Knowledge on general aspects of non-vaccine cold chain commodities

The knowledge on non-vaccine commodities cold chain storage and handling was assessed by investigating the knowledge on heat sensitivity of non-vaccine commodities cold chain, the use of a refrigerator for non-vaccine commodities, and the general aspect of non-vaccine commodities storage (Fig. 1A–C). The findings showed that most of the participants 47 (67.8%) were knowledgeable about the heat sensitivity of the cold chain commodities. However, fewer participants 30 (43.5%) had knowledge about the effect of freezing on non-vaccine commodities cold chain (Fig. 1A). Moreover, the majority number of participants showed less knowledge regarding the storage of non-vaccine commodities by the doorway 55 (80%) and central positioning of the thermometer 35 (50%) as well as less knowledge on refrigerator use for storage of non-vaccine commodities 33(48.1%) (Fig. 1B). In addition, 31 (45%) participants had inadequate knowledge regarding the use of general-purpose refrigerators while 38 (55%) were storing other items in the non-vaccine cold chain refrigerator (Fig. 1C).

## Discussion

The non-vaccine commodities cold chain forms a core part of diagnostic and curative interventions and their protection against loss of potency is key. This study assessed cold chain management practices of non-vaccine commodities in health centers of the Rwenzori Region, Western Uganda.

The findings of the study revealed that few health centers had stock cards for cold chain medicines correctly filled and conducted monthly physical inventory. This could be attributed to a lack of central stock control in the facilities since central or main stores lack refrigerators. Management of the cold chain among health center III and IVs in the Rwenzori sub-region is an issue given that only a few of the facilities had non-vaccine refrigerators as reported elsewhere [7]. In Uganda like in many African countries, better working refrigerators for cold chain are concentrated in the urban centers and transport terminals, such as airports [13]. However, they are needed given the terrain as Koskei et al. [14] stress the need for expensive but durable and suitable refrigerators for rural areas to cut down on repairs costs which often time attract huge costs due to transporting needs of technicians. Further, cold chain management in hospitals is very poor compared to that of other institutions; it is even worse in public than private health facilities [15].

More than half of refrigerators had temperature charts present with a recording chart available but 50% of HFs had a well-updated temperature chart creating a gap as all cold chain storage equipment should have the charts. This is not a good indicator as charts are fundamental in evidence based cold chain monitoring as reported in the literature [16]. Only a few health facilities had implemented safety measures like having switchless plugs and cautionary notices in the cold chain rooms or around cold chain facilities such as ‘‘do not unplug’’ implying that they used natural knowledge that is not sustainable and catastrophic. This was also reported to be used in a previous study carried out in Kenya on cold chain management practices [5]. Only 39% of the refrigerators observed had a sticker on the door to remind staff to open the door only when necessary. Besides, refrigerator servicing, defrosting and cleaning of the cold chain storage was not regularly conducted and yet frequent door opening promotes temperature excursions.

The majority of respondents had alternative plans regarding the malfunctioning of the refrigerators' lack of power backup or lack of refrigerators. Some of these alternatives implied the use of more resources in terms of frequent picking of the commodities from a far-off neighboring facility. This scenario is consistent with the findings of other previously conducted research [17]. The revelation of only 45% of HFs having adequate and sufficient cold boxes to maintain cold chain medicines is disadvantageous to the health sector since it attracts a loss of up to 55% of the medicines arising from poor maintenance of medicines in recommended temperature ranges of 2–8 degrees Celsius. This is common among developing economies [18]. This may be attributed to inadequate funding and lack of prioritization. Usually, this is associated with cooler boxes at the SDPs being in bad working conditions with damaged thermometers as was the case in Lamu county in Kenya [12]. Issues of cooler boxes go in hand with damaged fluid ice packs necessitating changing them daily as advised in the published literature [17] to secure recommendable potency levels.

The level of knowledge of the staff on cold chain storage and handling was generally adequate with gaps in specific aspects like implications of extreme heat or coldness on cold chain. This can be attributed to good training of the personnel while in school. Additionally, respondents had an adequate understanding of the effects of exposing the cold chain to sunlight and aspects positioning of the fridge on the wall and disposing of fridge contents once the fridge is switched off. This is due to the existence of a policy in a working environment as supported by previous research [5].

On storage of vaccines with non-cold chain vaccines, an average of 47(68%) knew that vaccines could not be safely stored with non-vaccine cold chain items such as lab reagents. Incidentally, this was not the practice on the ground. This is a case of not practicing what is known and is attributed to a lack of enforcement as supported by the literature [19]. The study revealed that respondents need training in power management aspects and that the capacity of management to supervise and enforce best practices needed to be boosted as well. These findings are in line with a study conducted in Ethiopia [17]. This study observed some knowledge gaps among staff managing cold chain commodities with specific reference to Oxytocin injection. Staff generally lack skills essential to effectively run the docket and thus need regular refresher trainings to build their capacity [20]. In fact, from the current study, its only a few staff who attended specialized training on cold chain management during their time of service. With a staff population of over 40% having worked for an average of over 7 years, the skills gap is glaring. Supervision was also not done frequently by the district and ministry of health. Supervision was done even rarely by health facility in-charges who are also overwhelmed with other management work. Many scholars assert that employees should get a refresher course after every 2 years at the minimum [5]. This is supported by Twinomujuni [21] who argues that because of new technologies and trends, new knowledge comes on board every other day and so to catch-up with this trend workers, need a routine on-job refresher course.

Backup was a challenge as is in most developing economies [18]. In fact, shortage of power is one of the major causes of losses and yet power outages are rampant. In absence of hydropower, other energy sources need to be in place to support a limited power supply with the objective to maintain the allowable temperature ranges. Despite (45%) of all the health facilities visited having generators, 80% were not found with fuel and are not automatic meaning that a significant delay can be experienced to either switch on the generator or even to fuel it in order to function. This condition is attributed to limited funding but also to a lack of technical support staff to effectively manage the power equipment. This is supported by the literature [12]. The same author argues that more than half of cold chain facilities are usually put to malfunction mode due to power-related issues. Therefore, whenever the main power source is disrupted, which is frequent in rural areas as reported in previous studies, everything goes to a standstill as the backup cannot play its role [4]. The lack of refrigeration equipment in the main stores implies poor central storage of stock and encroaching on EPI fridges overrunning WHO guidelines. The lack of central storage can compromise effective monitoring and stock control and the use of EPI refrigerators is against WHO cold chain management guidelines that aim to prevent medication errors and other undesired outcomes. Other facilities may decline to procure these commodities denying clients the opportunity to access health services provided by the use of these commodities. This challenge was also reported in a previous study that found that faulty equipment are common in public health facilities and most of them are due to negligence on the part of managers [22]. This problem cuts across most developing countries and many breakdowns are reported in public as compared to private health facilities [21].

The majority of the HFs did not have guidelines for cold chain storage management. This was seen as a weakness that requires attention. The proposal was that the existing guidelines should be reviewed with inputs from all stakeholders and they should be published and appropriately disseminated. It is argued that any organization without clear guidelines is bound to underperform on its mandate since guidelines set the parameters for who does what and how [23].

Based on the current results, the following are the recommendations. There is a need to entrench capacity building within the planning and budgeting processes of the health center IIIs and IVs and encourage a more participatory approach in developing new, reviewing existing guidelines as a way of making the cold chain staff part of the equation of cold chain management. Equipping health centers with in-good-condition refrigerators and recruiting qualified staff should be a priority for all stakeholders as they pose to be major challenges associated with the management of cold chain medicines.

### Study strengths, limitations and future research

This current study adds to the body of knowledge on the management of non-vaccine commodities which is lacking from the previous studies that focus on the cold chain management of vaccines.

Given that the study targeted health center IIIs and IVs, many of the health center IIIs had been recently elevated from health center IIs and as such did not have adequate infrastructure. More so, the study did not include hospitals and private health facilities. Therefore, there is a need for a similar study on cold chain management practices in general hospitals at public and private sectors to better inform cold chain management policy at the national level. In addition, a study to ascertain the quality of non-vaccine cold chain commodities used in Public Health Facilities is needed.

## Conclusion

The overall management practices for non-vaccine cold chain commodities were fair. The larger proportion of the health facilities lacked refrigerators and was using EPI fridges for storage of non-vaccine cold chain commodities. Most of the health facilities lacked power backup. More than a third of the facility staff lacked knowledge on cold chain management practices for non-vaccine commodities.

## Data Availability

Data sets and materials for information in this manuscript can be provided by the first author upon reasonable request.

## Abbreviations

CDC: Center for Disease Control
EPI: Expanded Program on Immunization
GDP: Gross domestic product
HC: Health center
HF: Health facility
MAKSHSREC: Makerere University School of Health Sciences Research and Ethics Committee
SDP: Service delivery point
SOP: Standard operating procedures
WHO: World Health Organization
